# miR-29a modulates SCD expression and is regulated in response to a saturated fatty acid diet in juvenile genetically improved farmed tilapia (*Oreochromis niloticus*)

**DOI:** 10.1242/jeb.151506

**Published:** 2017-04-15

**Authors:** Jun Qiang, Yi Fan Tao, Jie He, Yi Lan Sun, Pao Xu

**Affiliations:** Key Laboratory of Freshwater Fisheries and Germplasm Resources Utilization, Ministry of Agriculture, Freshwater Fisheries Research Center, Chinese Academy of Fishery Sciences, Wuxi, Jiangsu 214081, China

**Keywords:** GIFT, miR-29a, Stearoyl-CoA desaturase, Saturated fatty acid

## Abstract

MicroRNAs (miRNAs) are small non-coding RNAs that regulate target gene expression by binding to the 3′ untranslated region (3′ UTR) of the target mRNA. MiRNAs regulate a large variety of genes, including those involved in liver biology and disease. Here, we report for the first time that miR-29a post-transcriptionally regulates stearoyl-CoA desaturase (SCD) by binding to its 3′ UTR in genetically improved farmed tilapia (GIFT), *Oreochromis niloticus*, as shown by a 3′ UTR luciferase reporter assay. miR-29a antagomir treatment *in vivo* resulted in significant upregulation of SCD expression. We found that miR-29a expression was negatively correlated with SCD expression in GIFT liver. Inhibition of miR-29a led to a significant increase in SCD expression on day 60 induced by a saturated fatty acid diet, thereby increasing conversion of 16:0 and 18:0 to 16:1 and 18:1, respectively, and activating serum insulin, which would favor glucose and lipid uptake by the liver. These results indicate that miR-29a regulates SCD levels by binding to its 3′ UTR, and this interaction affects saturated fatty acid stress induction and insulin and lipid accumulation in serum. Our results suggest that miR-29a is critical in regulating lipid metabolism homeostasis in GIFT liver, and this might provide a basis for understanding the biological processes and therapeutic intervention encountered in fatty liver.

## Abstract (precis)

**Summary:** miR-29a targets SCD 3′ UTR directly. Inhibition of miR-29a could mediate conversion of C16:0 and C18:0 to C16:1 and C18:1, respectively, and activate serum insulin and glucose uptake in GIFT by increasing SCD.

## INTRODUCTION

Genetically improved farmed tilapia (GIFT), *Oreochromis niloticus* (Linnaeus 1758), is a very adaptable species in southern China, occurring in provinces such as Hainan, Guangxi, Guangdong and Fujian (Qiang et al., 2012). Increasing the amount of carbohydrate or lipid in their diet might help to improve growth and reduce feed cost (Tan et al., 2009). However, there is growing concern about fatty liver disease in GIFT fed high fat or high carbohydrate diets (Xiong et al., 2014; Qiang et al., 2016). Excess accumulation of lipid droplets within hepatocytes results in hepatic steatosis, which may cause metabolic dysregulation, reduce growth performance and impair both bone development and the oxidative response (Tan et al., 2009; Borén et al., 2013; Qiang et al., 2016). In particular, the accumulation of excess saturated fatty acids (SFAs) in liver of fish has been tied intimately to pathogenesis of hepatic lipid metabolism, such as accumulation of triglyceride (TG) and total cholesterol (TC), and oxidative stress (Hixson et al., 2016; Yu et al., 2012). In fish, the liver plays a crucial role in lipid homeostasis, which governs lipid synthesis, catabolism, storage and secretion (Zhang et al., 2014). Although the mechanism underlying SFA-induced metabolic dysregulation in fish liver is unclear, several studies in mammals have suggested that the effected fatty acid oxidation and insulin resistance resulted from the regulation of stearoyl-CoA desaturase (SCD) (Yokoyama et al., 2012; Miyazaki et al., 2009), insulin receptor (Yang et al., 2014a) and insulin receptor substrate (Yang et al., 2014b) modification and expression. SCD is an enzyme involved in the biosynthesis of monounsaturated fatty acids (MUFAs), which play an important role in the regulation of hepatic lipid metabolism (Dobrzyn and Ntambi, 2004; Ntambi and Miyazaki, 2004). SCD plays a key role in introducing the first double bond between carbons 9 and 10 of palmitoyl (16:0)-CoA and stearoyl (18:0)-CoA to form monounsaturated palmitoleic acid (16:1) and oleic acid (18:1), respectively (Heinemann and Ozols, 2003). High expression of SCD1 was found in adipose and liver tissue of mouse. Repressed SCD1 expression in mice resulted in hyperphagia, but mice were lean and reduced obesity induced by a high lipid diet (Miyazaki et al., 2009). Additionally, the tolerance of glucose and insulin in whole body was stimulated in SCD1-null mice (Miyazaki et al., 2009).

MicroRNAs (miRNAs) are small non-coding, single-stranded RNAs (18–25 nucleotides) found in plants, animals and some viruses that function in RNA silencing and post-transcriptional regulation of gene expression by binding to the 3′ untranslated region (3′ UTR) of their target mRNAs (Bazzini et al., 2012; Pillai et al., 2007). miRNAs play extremely important roles in regulating gene expression and have been reported to be instrumental in mediating liver biology and disease in mammals (Bandiera et al., 2015; Bhatia et al., 2014). The miR-29 family is one of the best-known miRNA families, which has been associated with intermediate lipid metabolites such as ceramide and diacylglycerol (Yang et al., 2014b; Mattis et al., 2015). In C57BL/6 mice fed the SFA palmitate and a high fat diet, miR-29a was induced and downregulated the mRNA expression level of insulin receptor substrate-1 (IRS-1) to regulate insulin signaling in myocytes (Yang et al., 2014b).

In addition, inhibition of adipogenic gene expression and impaired accumulation of lipid in mice hepatocytes were induced by miR-29a regulation, which plays a key role in energy metabolism of mice fed a high fat diet (Yang et al., 2014b). However, the detailed mechanism of hepatic steatosis in GIFT by the miR-29a regulator remains unclear.

List of symbols and abbreviations

3′ UTR: 3′ untranslated region
ALT: alanine aminotransferase
AST: aspartate aminotransferase
*C*_t_: threshold cycle
GIFT: genetically improved farmed tilapia
HFD: high fat diet
HSI: hepatopancreas somatic index
IRS-1: insulin receptor substrate-1
MG: mass gain
miRNA: microRNA
MUFA: monounsaturated fatty acid
NFD: normal fat diet
qRT-PCR: quantitative real-time RT-PCR
SCD: stearoyl-CoA desaturase
SFA: saturated fatty acid
SGR: specific growth rate
TC: total cholesterol
TG: triglyceride
UFA: unsaturated fatty acid

We conducted this study to evaluate the involvement of miR-29a in GIFT lipid metabolism. We identified for the first time the SCD 3′ UTR as a putative target of miR-29a through RNA-seq screening. Expression analysis by quantitative real-time RT-PCR (qRT-PCR) further revealed the miR-29a-mediated control of SCD in GIFT fed SFA diets. The present work suggests a novel mechanism whereby miR-29a is related to the development of hepatic steatosis in GIFT, and provides an improved understanding of lipid metabolism and the mechanisms involved in fatty liver disease for possible therapeutic intervention.

## MATERIAL AND METHODS

### Ethics statement

The study protocols were approved by the Freshwater Fisheries Research Center at the Chinese Academy of Fishery Sciences (Wuxi, China). The fish were kept in well-aerated water and were anesthetized by injecting 0.01% tricaine methanesulfonate (Sigma-Aldrich, St Louis, MO, USA) before sampling, and the livers were extracted based on the Guide for the Care and Use of Laboratory Animals in China.

### Small RNA library construction and verification

Healthy GIFT from the Yixing tilapia farm of the Freshwater Fisheries Research Center, Chinese Academy of Fishery Sciences, were selected as the experimental fish. Twenty-five GIFT were fed high fat diets (17% fat diet; HFD) as the stress group, and another 25 fish were fed normal fat diets (6.8% fat diet; NFD) as the normal group (Table S1). Feed was offered to apparent visual satiety two times per day (08:00 and 15:00 h). Continuous aeration was applied during the 30-day experiment. Feces were removed daily using a siphon, and approximately 20% of the water was replaced every 3 days, with a temperature difference <±0.3°C. Dissolved oxygen was maintained at ≥6 mg l^−1^, and pH was 7.4–7.8. Ammonia-N and nitrite concentrations were both <0.01 mg l^−1^, and the photoperiod was 12 h:12 h light:dark. At the end of the trial, liver tissues from nine fish (NFD group or HFD group) were collected separately and immediately stored in liquid nitrogen at −80°C for transcriptome sequencing and expression analysis. The samples, which contained equal amounts of RNA extracted from the liver tissues from the HFD and NFD groups, were mixed and pooled to construct the miRNA libraries by high-throughput sequencing on a HiSeq 2000 system (Illumina, San Diego, CA, USA). Low quality reads and reads contaminated with adapter sequences were removed before analysis (Xu et al., 2015). The filtered sequences (18–30 nt) were aligned to the zebrafish (*Danio rerio*) genome sequence using the short oligonucleotide alignment program (SOAP; http://soap.genomics.org.cn) with a tolerance of one mismatch.

To determine the differential expression levels of the miRNAs between the HFD and NFD libraries, the sequencing data were normalized (Zhu et al., 2010) and the fold change was determined as: fold change=log 2(HFD/NFD). The data from the two libraries were statistically analyzed based on the Audic and Claverie method (Audic and Claverie, 1997). The differential expression levels of miRNA were verified by qRT-PCR.

### miR-29a targets prediction and tissue distribution in GIFT

We used the Nile tilapia transcriptome data (https://www.ncbi.nlm.nih.gov/genome/?term=Oreochromis+niloticus) and the miRanda v3.01 toolbox to predict target genes that contained a single candidate site in the 3′ UTR for the miRNAs. Targets identified using the default parameters and cut-offs (score *S*≥140; energy *E*≤−7.0 kcal mol^−1^) (Betel et al., 2010) and related to lipid metabolism were selected as promising candidate genes. Six tissues (muscle, liver, kidney, spleen, blood and gut) from healthy GIFT were selected and the miRNA tissue distribution was determined by qRT-PCR.

### RNA preparation and qRT-PCR

miRNAs were extracted using an amiRNeasy kit (Takara, Dalian, China) according to the manufacturer's protocol and a Mir-X™ miRNA First-Strand Synthesis kit (Takara) was used to synthesize the first-strand cDNA. The 10.0 μl reverse transcription (RT) reaction mixture contained 5.0 μl 2× mRQ buffer, 3.75 μl RNA sample (0.25–8 μg) and 1.25 μl mRQ enzyme. The reactions were incubated at 37°C for 60 min, 85°C for 5 min and held at 4°C. The qRT-PCRs were performed using an miRNA SYBR Green qRT-PCR Kit (Takara) with the provided miRNA reference gene (U6). The 25 μl PCR contained 2.0 μl of the RT product (template), 12.5 μl 2× SYBR Advantage Premix, 9 μl ddH_2_O, 0.5 μl 50× ROX Dye, 0.5 μl miRNA-specific primer (10 μmol l^−1^) and 0.5 μl mRQ 3′ primer. The default thermal profile used for PCR amplification consisted of 95°C for 10 s, followed by 40 cycles of 95°C for 5 s and 60°C for 20 s, with a final dissociation curve at 95°C for 60 s, 55°C for 30 s and 95°C for 30 s. Dissociation curve analysis of amplified products was performed at the end of each PCR reaction to confirm that only one PCR product was amplified and detected. For each cDNA, three-well replicates were used. The threshold cycle (*C*_t_) value was determined using the automatic setting on the ABI 7900HT Fast Real-Time PCR system (Applied Biosystems, Foster City, CA, USA). *C*_t_ values determined for each sample were normalized against the values for U6. The relative fold change in expression to U6 was calculated by the 
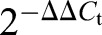
 method (Livak and Schmittgen, 2001), and the values related to the control group represented the *n*-fold difference. In order to detect the presence of non-specific amplifications, control reactions without template were included for each primer set. The miR-29a-specific primer (5′-GCACCATTTGAAATCGGTTAG-3′) was synthesized by Genewiz (Suzhou, China).

Total RNA was isolated with TRIzol Reagent (Invitrogen, Carlsbad, CA, USA). PrimeScript™ RT Master Mix (Takara) was used for the RT reaction of the miRNA target genes. The 10.0 μl RT reaction contained 2.0 μl 5× PrimeScript RT Master Mix, RNA sample (≤500 ng) and RNase Free dH_2_O up to 10 μl. The reactions were incubated at 37°C for 15 min, 85°C for 5 s and held at 4°C. The qRT-PCRs were analyzed using SYBR^®^ Premix Ex Taq kits. The 20 μl PCR included 2.0 μl of the RT product, 10.0 μl 2× SYBR^®^ Premix Ex Taq II, 0.8 μl each of PCR forward and reverse primers (10 μmol l^−1^), 0.4 μl 50× ROX Dye and 6 μl ddH_2_O. The reactions were incubated at 95°C for 30 s followed by 40 cycles of 95°C for 5 s and 60°C for 30 s. The 18S rRNA transcript level was taken as a reference to calculate the relative expression level of each target gene. The mRNA primers for SCD were F: 5′-ACAAGCTCTCCGTGCTGGTCAT-3′, R: 3′-GCAGAGTTGGGACGAAGTAGGC-5′ and for 18S rRNA they were F: 5′-GGCCGTTCTTAGTTGGTGGA-3′ and R: 5′-TTGCTCAATCTCGTGTGGCT-3′. The primers were synthesized by Shanghai GeneCore Bio Technologies Co. (Shanghai, China). The mRNA expression levels were analyzed using the 
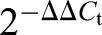
 method (Livak and Schmittgen, 2001), and the values related to the control group represented the *n*-fold difference. The mRNA expression levels were quantified using an ABI 7900HT Fast Real-Time PCR System (Applied Biosystems) and compared using Relative Quantification (RQ) manager software.

### SCD 3′ UTR luciferase reporter assay

HEK 293T (human embryonic kidney) cells were obtained from the Shanghai Bioleaf Biotech Co. (Shanghai, China). The cells were maintained in Dulbecco's modified Eagle's medium with 10% fetal bovine serum, 100 U ml^–1^ penicillin, 100 μg ml^–1^ streptomycin and 250 ng ml^–1^ amphotericin B, and maintained at 37°C in a humidified 5% CO_2_ incubator. The reagents were purchased from Sigma-Aldrich.

The SCD 3′ UTR luciferase reporter vector was constructed for analyzing the potential miRNA target sites and the effect of miR-29a on its activity was evaluated in the HEK293T cell line. The full-length 3′ UTR from SCD was chemically synthesized and inserted downstream of the luciferase gene in the pGL3-control vector (Promega, Madison, WI, USA). To construct the pGL3-SCD mutant, six base pairs (5′-GTGCTA-3′) in the SCD 3′ UTR region were deleted and six new base pairs (5′-AGCTAC-3′) were inserted. Synthetic miRNA mimics were synthesized by RiboBio (Guangzhou, China) as RNA duplexes designed from the miR-29a sequence (5′-CUAGCACCAUUUGAAAUCGGUUA-3′) in the miRBase database. A scrambled miRNA mimic (5′-UUUGUACUACACAAAAGUACUG-3′) with no homology to the tilapia genome was used as the negative control. Twenty-four hours prior to transfection, HEK 293T cells were plated at 1.0×10^6^ cells per well in 12-well dishes. Cells were transfected with 25 ng firefly luciferase reporter vector containing either the wild-type or mutant 3′ UTR constructs, with or without 50 nmol l^−1^ miR-29a mimic or negative control and 5 ng *Renilla luciferase* control vector (pRL-TK, Promega), using Dharma FECT Duo (Thermo Scientific Dharmacon). Firefly luciferase activity was normalized to *Renilla luciferase* activity for each transfected well. Then, 36 h after transfection, the cells were washed with ice-cold PBS and centrifuged at 800 ***g*** at 4°C for 5 min to harvest the cells. A liquid scintillation counter (Hitachi, Japan) was used to detect luciferase activity with a standard dual-luciferase reporter system according to the manufacturer's instructions (Krek et al., 2005). The assay kits were purchased from Shanghai Lengton Bioscience Co. (Shanghai, China). Five biological replicates were performed for each treatment.

### Functional analysis of miR-29a *in vivo*

A chemically modified antisense oligonucleotide (miR-29a antagomir: 5′-UAACCGAUUUCAAAUGGUGCUAG-3′) was synthesized to regulate miR-29a expression (RiboBio). The 3′ end of the oligonucleotide was conjugated to cholesterol, and all the bases were 2′-OMe modified. The antagomir oligonucleotide was deprotected, desalted and purified by high-performance liquid chromatography. To analyze the silencing miR-29a expression levels, 270 juvenile GIFT weighing approximately 4.5 g were distributed randomly to nine 600-liter tanks, each containing 30 fish. The fish were tail-vein injected with miR-29a antagomir, negative antagomir (four mismatch mutations in each miRNA sequence) or the same volume of PBS at a dose of 50 mg kg^−1^ body mass every 3 days for 21 days. The PBS-treated fish were taken as the control (Yan et al., 2013a,b). Livers were sampled from three fish from each tank at the following time periods: 0, 7, 14 and 21 days. The liver tissues were collected, immediately frozen in liquid nitrogen and stored at −80°C until used for qRT-PCR. At the end of the trial, another three fish were selected randomly from each tank. Liver samples were obtained and store for analyses of biochemistry and enzyme activities. After collecting the samples, the mass gain (MG) and specific growth rate (SGR) of the 10 fish per tank were measured as:

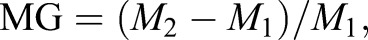


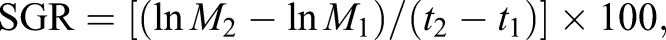
where *M*_1_ and *M*_2_ are body mass (g) at the start (*t*_1_) and end (*t*_2_) of the experimental period.

### SFA-regulated trial

The feeding trial was performed in a water recirculating system comprising six plastic tanks (800 liters each) maintained at 29±0.3°C. GIFT were fed either a control diet (5% fish oil) or an SFA diet (SFA, 5% coconut oil) (Table S2). Approximately 600 liters of treated water (aerated for three consecutive days) were added to each of the six plastic tanks, and 30 fish per tank were fed each experimental treatment (each treatment had three replicates). At the beginning of this experiment, 30 fish were taken in triplicate from a common tank to record initial masses. Wet mass was recorded using an electronic digital balance (±0.01 g), and the initial mean mass of each juvenile was 2.78±0.11 g. Results of a multivariate ANOVA showed no differences in mass between fish in the different treatments and replicate treatments (*P*>0.05). Rearing management was the same as that used in the experiment of HFDs.

Feeding was stopped 24 h prior to collecting the samples. Three fish were collected from each tank on days 20, 40 and 60 during the experiment, and liver samples were obtained, frozen immediately in liquid nitrogen and stored at −80°C until mRNA levels and fatty acid compositions were measured. At the end of the trial, six fish were selected randomly from each tank. Blood samples were collected from the caudal vein of three fish from each tank. All the blood samples were kept at 4°C for 2 h and then centrifuged at 4°C and 3500 ***g*** for 10 min to collect the serum, which was stored at −80°C for later use. Liver samples were obtained from the remaining three fish, immediately frozen in liquid nitrogen and stored at −80°C for later biochemistry analyses. Finally, liver tissue blocks of six fish from two treatments (one fish from each of six tanks) were washed with physiological saline and fixed with Bouin's solution and 2.5% glutaraldehyde solution for 24 h in order to prepare paraffin sections, which were examined using an optical microscope.

After collecting the samples, the body and liver masses of the remaining fish were measured as: MG, SGR and hepatopancreas somatic index [%; HSI=(liver mass × 100)/*M*_2_].

### Serum and hepatic biochemical analyses

The levels of serum glucose, TG and TC were measured using a Roche Cobas C311 automatic biochemical analyzer (Roche, Basel, Switzerland). Serum alanine aminotransferase (ALT) and aspartate aminotransferase (AST), and hepatic TG, TC and glycogen were determined by enzyme-linked immunosorbent assay (ELISA) using test kits. All assay kits used were from Shanghai Lengton Bioscience.

Serum insulin levels were measured by radioimmunoassay using bonito insulin as the standard and rabbit anti-bonito insulin as antiserum, according to the method described by Gutiérrez et al. (1984). The minimum detection limit was 0.15 mIU l^−1^, with intra- and inter-assay coefficients of variation of 4.4% and 10.3%, respectively (*n*=9).

### Fatty acid analysis

Total lipids were extracted from liver tissue and fatty acid analyses were conducted according to the methods described previously (He et al., 2015).

### Statistical analysis

Statistical analysis of data was performed by two-way ANOVA using SPSS 17.0 (SPSS Inc., Chicago, IL, USA). Each value represents nine replicates. Data were tested for normality and homogeneity of variances, and log transformed when necessary. When the effects of sampling times and/or type of lipid sources were significant, the two factors were analyzed separately by one-way ANOVA. A *P*-value <0.05 was considered significant. Signiﬁcant differences in different treatments at each sampling point were calculated with Duncan's multiple range tests. Signiﬁcant differences between values obtained at the different sampling times were calculated with paired-sample *t*-tests.

## RESULTS

### Identification of miR-29a expression and target gene

**Fig. 1.**
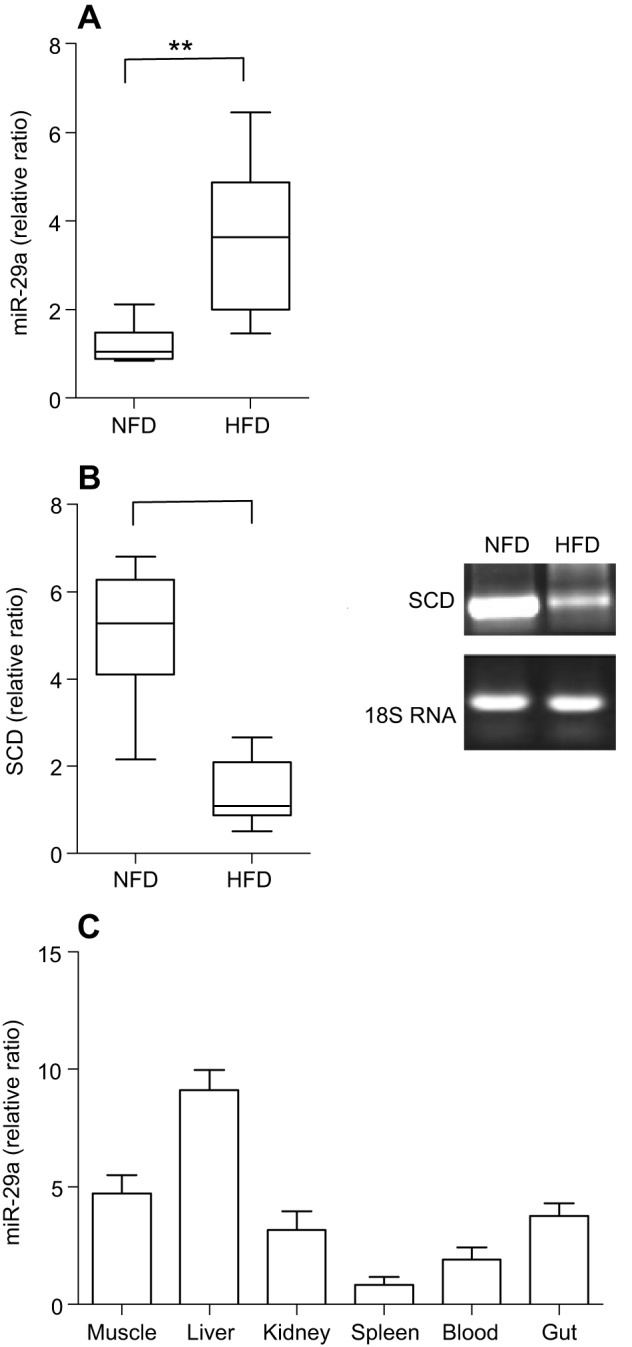
**Regulation of miR-29a and stearoyl-CoA desaturase (SCD) by normal fat diet (NFD) and high fat diet (HFD) groups and expression pattern of miR-29a in genetically improved farmed tilapia (GIFT) extracted from different tissues (*n*=9 replicates per group).** (A) The expression of miR-29a was quantified by qRT-PCR from NFD and HFD in GIFT liver. The values are expressed as the relative ratio with U6 as reference gene. **P<0.01. (B) The expression of SCD was quantified by qRT-PCR from NFD- and HFD-fed GIFT tilapia liver. Values are expressed as the relative ratio with 18S rRNA as the reference gene. (C) Expression pattern of miR-29a in GIFT. miRNA samples were extracted from different tissues, including muscle, liver, kidney, spleen, blood and gut. miRNA expression was detected by qRT-PCR.

**Fig. 2.**
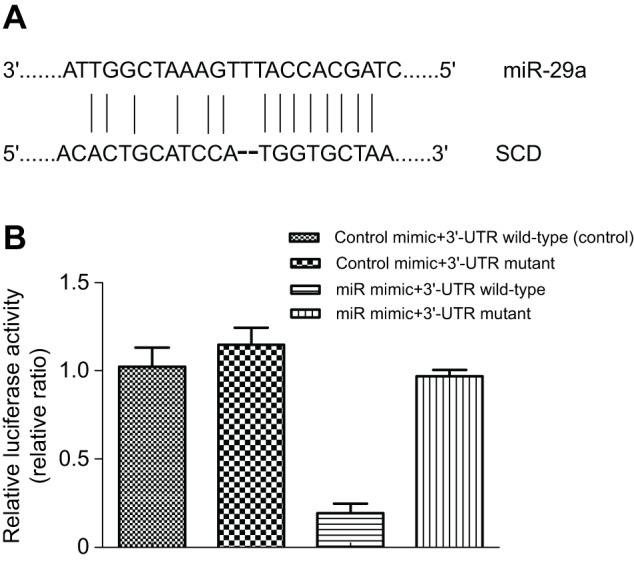
**miR-29a regulated SCD gene expression by binding with SCD 3′ UTR.** (A) Sequence alignment of miR-29a binding sites with SCD 3′ UTR. (B) The SCD 3′ UTR luciferase reporter vector was constructed and the miR-29a–SCD binding sites in the HEK293T cell lines were evaluated by the dual-luciferase reporter system. The treatment of control mimic+3′-UTR wild-type in HEK293T cell lines was taken as the control group.

Through deep sequencing, we found that the expression levels of certain miRNAs, such as miR-34a, miR-145-5p, miR-29a and miR-23a-3p, increased in the liver of the GIFT fed the HFD compared with the NFD (Table 1). The upregulated expression of miR-29a in the HFD group compared with the NFD group was verified to be significant by qRT-PCR (*P*<0.05; Fig. 1A). In this study, we focused on the regulatory role of miR-29a in lipid metabolism of GIFT. Four putative targets of miR-29a were identified by screening the RNA-seq data with miRanda v3.01, namely, very-long-chain 3-oxoacyl-CoA reductase-B-like, ELOVL fatty acid elongase 6, SCD and lipase member H-like. A region of the 3′ UTR of SCD completely matched (from 3812 to 3833 bp) the seed sequence of miR-29a (Fig. 2A), and SCD had the highest score (153) and lowest free energy (−16.07 kcal mol^−1^) among the candidate target genes. We found a significant decrease in the SCD expression levels in the HFD group compared with the NFD group (Fig. 1B). We then used qRT-PCR to detect the tissue distribution of miR-29a (Fig. 1C). The result shows that miR-29a was expressed mainly in muscle, liver, kidney and gut, all of which are involved in the regulation of lipid metabolism. Fish liver is involved in a wide variety of vital functions that require highly orchestrated and controlled biochemical processes. Therefore, in this study we mainly investigated the relationship between miR-29a and SCD in liver of GIFT.

**Table 1.**
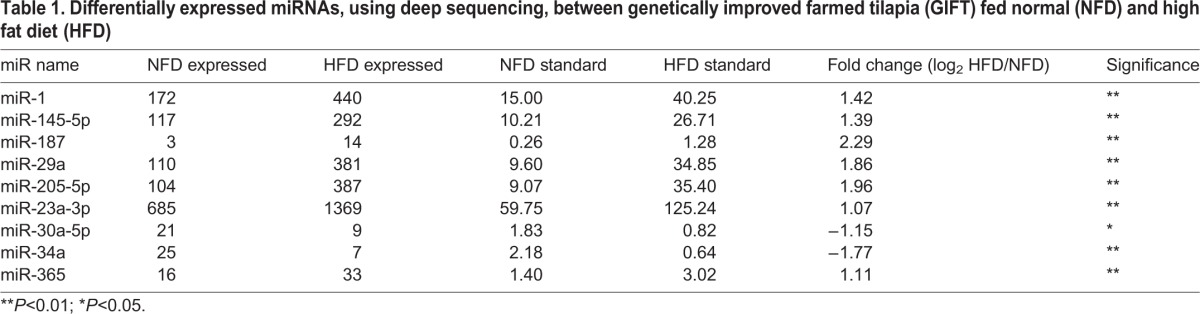
**Differentially expressed miRNAs, using deep sequencing, between genetically improved farmed tilapia (GIFT) fed normal (NFD) and high fat diet (HFD)**

### miR-29a acts directly at the 3′ UTR of SCD

To verify that miR-29a directly inhibits SCD expression, we employed a luciferase reporter assay. The alignment of miR-29a with the SCD 3′ UTR is shown in Fig. 2A. We constructed two different luciferase reporters, namely the wild-type SCD 3′ UTR and a mutant SCD 3′ UTR that lacked the miR-29a binding site (Fig. 2B). These luciferase reporters were cotransfected with miR-29a mimic into HEK293T cells. The results showed that the miR-29a mimic significantly decreased the luciferase activity of wild-type SCD 3′ UTR, while it did not affect the luciferase activity of mutant SCD 3′ UTR, suggesting that miR-29a directly suppressed SCD expression by binding to its 3′ UTR sequence.

### Effect of miR-29a inhibition on miRNA expression and hepatic TC and TG levels in juvenile GIFT

**Fig. 3.**
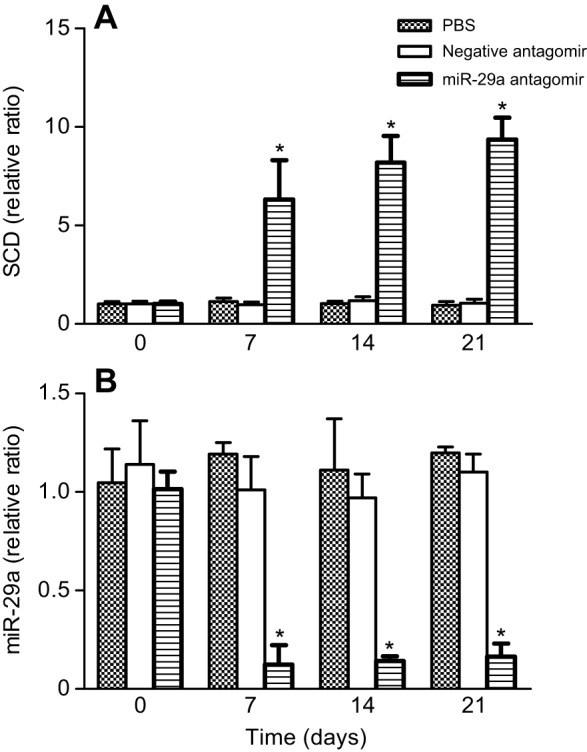
**Effect of miR-29a inhibition on miRNA and SCD expression in GIFT.** GIFT juveniles weighing ∼4.5 g were received a tail-vein injection of PBS, negative antagomir (four mismatch mutations in each miRNA sequence) or miR-29a antagomir at a dose of 50 mg kg^−1^ body mass every 3 days. The relative expression of SCD (A) and miR-29a (B) was detected using real-time PCR. The data were expressed as the relative change compared with the PBS group. *P<0.05.

In addition, we employed the antagomir method to determined miRNA loss of function *in vivo* (Fig. 3A,B). miR-29a antagomir treatment resulted in a significant reduction in miR-29a expression, while SCD expression was significantly upregulated at the different time points. No decrease in miR-29a or SCD expression levels was detected in GIFT injected with equal volumes of PBS or negative antagomir. These results suggest that miR-29a is involved in the regulation of SCD expression *in vivo*. After the 21-day injection trial, the growth and hepatic TG and TC were significantly higher in the miR-29a antagomir group compared with in the control and negative antagomir groups (*P*<0.05; Table 2).

**Table 2.**
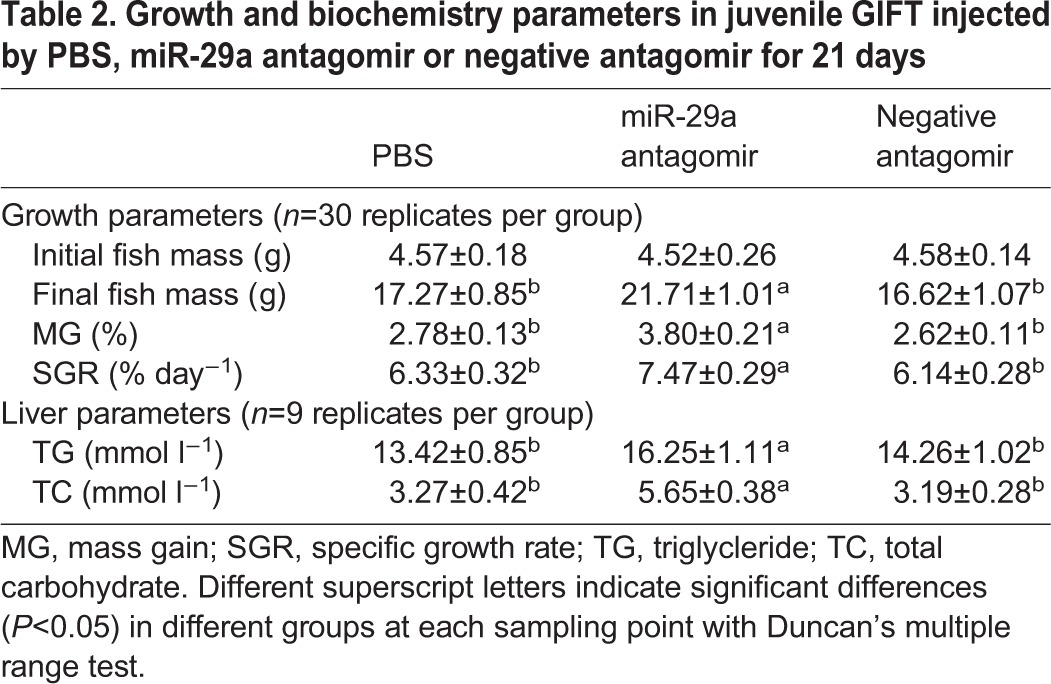
**Growth and biochemistry parameters in juvenile GIFT injected by PBS, miR-29a antagomir or negative antagomir for 21 days**

### Effect of SFA on growth and serum biochemical parameters in juvenile GIFT

After feeding GIFT with either the control diet (containing highly unsaturated fatty acid enriched by the addition of 5% fish oil) or the SFA diet (containing SFA enriched by the addition of 5% coconut oil) (Table S3) for 60 days, we found that the dietary lipid source significantly affected growth performance of the juveniles. Fish fed the SFA diet had less mass gain and a lower SGR compared with fish fed the control diet (Table 3). The HSI did not vary between the dietary treatment groups (*P*>0.05). In addition, the serum insulin, TC and TG levels in the SFA group were significantly higher than in the control group (*P*<0.05; Table 4). Serum glucose and high-density lipoprotein cholesterol levels showed no significant differences between the two groups, whereas serum low-density lipoprotein cholesterol levels were significantly higher in the SFA group compared with in the control group (*P*<0.05). Serum ALT and AST activities were not significantly different between the two groups (*P*>0.05). After the 60-day feeding trial, hepatic glycogen, TC and TG levels showed no significant differences (*P*>0.05; Table 4). No mortality was observed throughout the experiment.

**Table 3.**
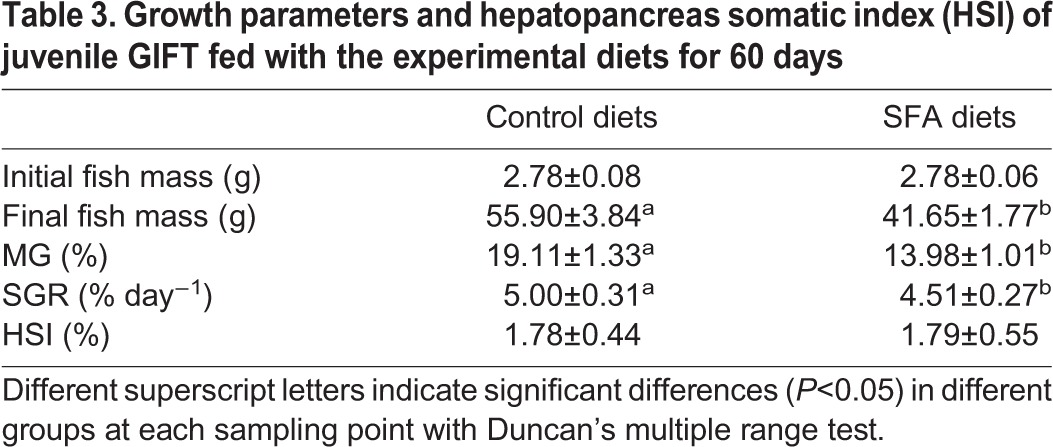
**Growth parameters and hepatopancreas somatic index (HSI) of juvenile GIFT fed with the experimental diets for 60 days**

**Table 4.**
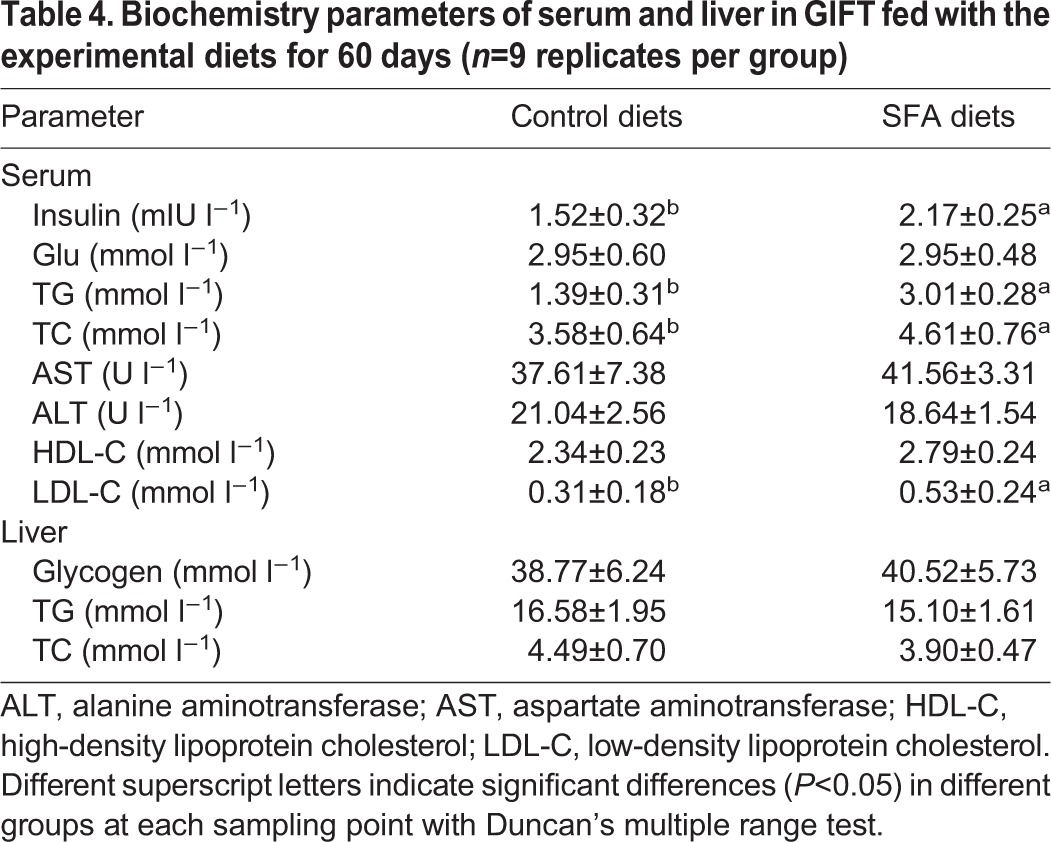
**Biochemistry parameters of serum and liver in GIFT fed with the experimental diets for 60 days (*n*=9 replicates per group)**

### Effect of SFA on liver tissue structure using paraffin sections from juvenile GIFT

**Fig. 4.**
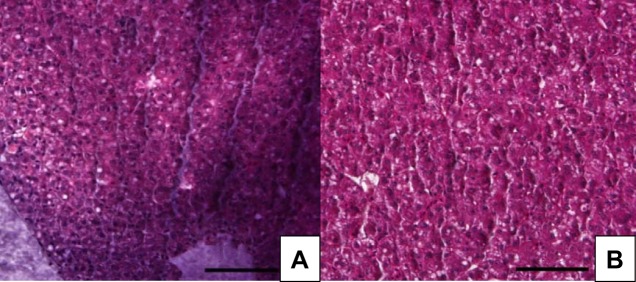
**Effects of different lipid sources on the liver structure of GIFT.** (A) Control diets group; (B) SFA diets group. A representative image is shown. Scale bar, 50 μm.

The structure of the hepatic cells in samples from the control group and the SFA group (Fig. 4) showed integrity and were distinct. The cells were arranged neatly and closely, and the cell nuclei were clear and showed integrity. In the liver tissue samples from the SFA-fed fish, accumulation of fat was rarely observed.

### Effect of SFA on miR-29a and SCD expression in liver from juvenile GIFT

**Fig. 5.**
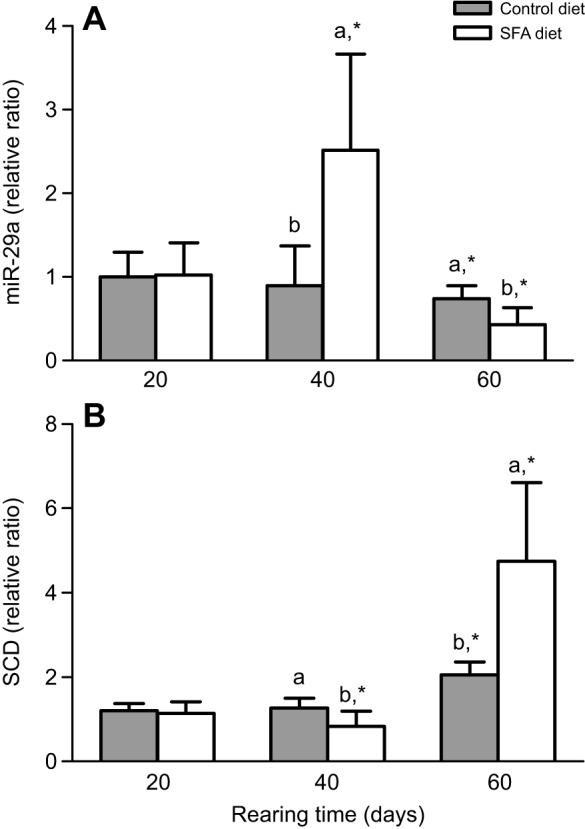
**miR-29a and SCD expression in response to different lipid sources (*n*=9 replicates per group).** GIFT were fed SFA or control diets. (A) The expression of miR-29a in liver was detected on days 20, 40 and 60 using qRT-PCR, with U6 as the reference gene. (B) The expression of SCD in liver was detected on days 20, 40 and 60 using qRT-PCR, with 18S rRNA as the reference gene. The group exposed to control diets on day 20 was taken as the control group. The data were expressed as the relative change compared with the control group on day 20. Asterisks indicate signiﬁcant differences (*P<0.05 paired-sample *t*-test) in each group among sampling points. Different superscript letters indicate signiﬁcant differences (*P*<0.05) in different groups at each sampling point with Duncan's multiple range test.

Hepatic miR-29a expression were significantly (*P*<0.05) influenced by dietary lipid sources and sampling time (Table S4, Fig. 5A). miR-29a expression was higher after 40 days than after 20 days in liver of fish fed the SFA diet. Hepatic miR-29a expression in the SFA and control groups showed significant decreases on day 60 compared with day 20. However, hepatic SCD expression was higher in the control and SFA groups on day 60 compared with days 20 or 40, and SCD expression was significantly higher on day 60 in the SFA group compared with the control group (*P*<0.05; Fig. 5B).

### Effect of SFA on fatty acid composition of liver from juvenile GIFT

**Fig. 6.**
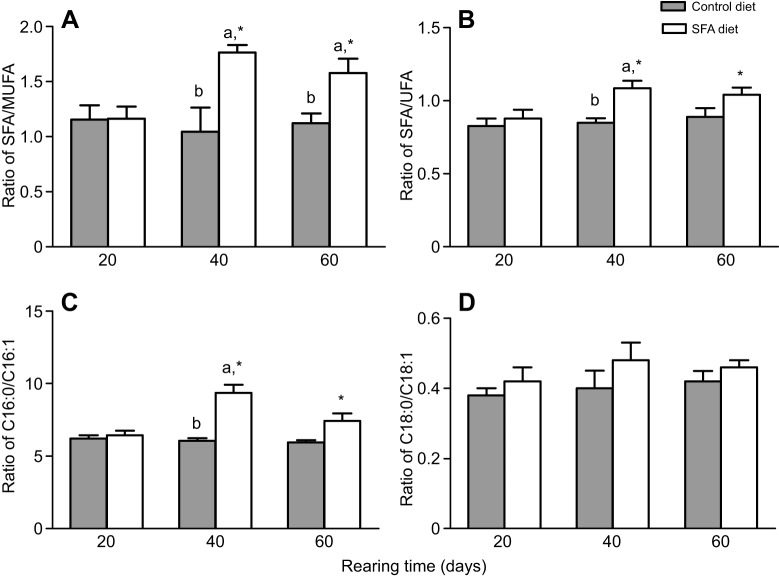
**Fatty acid ratios of GIFT tilapia in response to SFA or control diets on days 20, 40 and 60.** (A) SFA/MUFA, (B) SFA/UFA, (C) C16:0/C16:1 and (D) C18:0/C18:1. Asterisks indicate signiﬁcant differences (*P<0.05 paired-sample *t*-test) in each group among sampling points. Different superscript letters indicate signiﬁcant differences (*P*<0.05) in different groups at each sampling point with Duncan's multiple range test.

Liver fatty acid composition was affected by dietary lipid sources. Fish fed SFA diets exhibited significantly higher SFA/UFA (unsaturated fatty acid) and SFA/MUFA ratios (Fig. 6A,B) on day 40 than fish fed control diets (*P*<0.05), and fish fed the SFA diets showed a higher ratio of C16:0/C16:1 on day 40 than on day 60 (Fig. 6C). The fish fed the SFA diet had a slightly higher ratio of C18:0/C18:1 on day 40 than on day 20, and there were no significant differences in C18:0/C18:1 between day 60 and 20 (*P*>0.05; Fig. 6D).

## DISCUSSION

Increasing SCD activity stimulates lipid accumulation, but inhibited SCD would improve lipid utilization and impair the accumulated lipid in the liver (Flowers and Ntambi, 2008). SCD deficiency in mice caused upregulation of insulin-signaling components and affected glycogen metabolism in insulin-sensitive tissues (Hyun et al., 2010). We observed a remarkable decrease in SCD mRNA levels in GIFT fed HFD (Fig. 1B) and suspected that the post-transcriptional repression of SCD expression might occur via a miRNA-mediated mechanism. We found that the expression of SCD was increased in GIFT by injection of miRNA antagomir (Fig. 3A) and suggested that inhibited miR-29a increased SCD expression directly through translational repression (Fig. 3B), thereby regulating lipid metabolism in liver (Table 2). Furthermore, miR-29a was regulated in liver of GIFT fed the SFA diet (Fig. 5). Therefore, miR-29a may have a novel function in lipid metabolism of tilapia, and the induction of miR-29a by SFA may be involved in excessive lipid accumulation in blood.

The miR-29 family comprises three mature miRNAs – miR-29a, miR-29b and miR-29c – and their binding sites are highly conserved in humans and mice (Liston et al., 2012). In our study, we found that the miR-29a binding site (5′-TAGCACCA-3′) was well conserved in GIFT. Based on expression profiling and target validation studies by ectopic expression, the aberrant expression of miR-29a was reported to be associated with multiple biological processes, including insulin signaling and glucose metabolism, cell proliferation and differentiation, and immune modulation (Liston et al., 2012; Wang et al., 2013; Mattis et al., 2015). Recently, miR-29a was reported to target IRS-1 and was shown to be involved in glucose metabolism in the skeletal muscle of C57BL/6 mice (Yang et al., 2014a,b). However, little is known about the roles and functional significance of miR-29a in lipid metabolism and hepatic steatosis in fish. In the present study, we showed that a SFA diet affected the expression of miR-29a in the liver of GIFT on days 40 and 60, suggesting that miR-29a may be actively involved in the development of lipid metabolism. Moreover, miR-29a expression increased significantly in the liver of fish fed SFA on day 40, suggesting that an increase in miR-29a may be associated with response to SFA stress. Significantly increased miR-29a expression in a mouse model of diet-induced obesity or obese-type diabetic Goto-Kakizaki rats indicated that miR-29a is responsible for the development of obesity-induced insulin resistance and type 2 diabetes (Yang et al., 2014a,b; He et al., 2007; Karolina et al., 2011).

No continuous changes in the regulation of miR-29a were found, but the expression level of miR-29a was downregulated in fish fed SFA on day 60. In fish fed the normal diet, the change of miR-29a expression maintains the physiological responses to lipid or glucose metabolism. After being on the SFA diet for 60 days, miR-29a was downregulated, suggesting adaptive changes may have occurred in lipid metabolism homeostasis of GIFT liver. In fact, previous studies have revealed that miRNAs function as ‘on–off’ switches to regulate physiological or pathological processes, especially in long-term processes of biochemical metabolism (Williams et al., 2009; Du et al., 2010).

miRNAs are endogenous regulators of gene expression. They bind to specific mRNA targets, causing their degradation or translational repression (Bartel, 2009). A region of the SCD 3′ UTR completely matched a 7-nt ‘seed sequence’ at the 5′ end 2–8 site of miR-29a. We demonstrated for the first time that the antagomir of miR-29a increased SCD expression by directly targeting the SCD 3′ UTR (Fig. 3). Our results further suggest that downregulation of miR-29a might be triggered by SFA, resulting in upregulated transcriptional activation of SCD in response to lipid metabolism. SCD plays a crucial role in regulating fatty acid composition in membrane lipids and is also a key modulator in fatty-acid-mediated processes, including glucose homeostasis and lipid metabolism. SCD mRNA levels are stimulated by excess SFAs, which activate the SCD promoter (Ntambi and Miyazaki, 2004). Our results showed that SCD mRNA levels were higher in the liver of GIFT fed coconut oil compared with those fed fish oil on day 60. The fish oil diet had the composition of polyunsaturated fatty acids containing n-3 and n-6 fatty acids that is essential for maximal growth of GIFT (Table 3). The expression of SCD transcription may have increased in the tilapia after 60 days under the SFA diet to convert SFAs (16:0 and 18:0) to UFAs (16:1 and 18:1) for acclimation (Fig. 6) and to stimulate lipid and glucose transport. Hyun et al. (2010) reported that adipose-specific deletion of SCD1 leads to the reduction of adiponectin expression in adipose tissue, causing the upregulation of GLUT1 expression for maintenance of glucose transport in mice. The influence of SCD in lipid metabolism might differ according to the species, endogenous regulators and stress conditions. In addition, the change of miR-29a and SCD in the control fish (Fig. 5) might reflect the age of the fish or nutrition-regulated conditions as rearing time progressed.

The SCD gene has been shown to be positively regulated by insulin, the liver X receptor, and numerous dietary and cellular factors, including glucose, fructose and SFA (Dobrzyn et al., 2015). SCD1 affects insulin-signaling components and regulates lipid and glycogen utilization in metabolism-sensitive tissues of mice (Dobrzyn et al., 2005; Dobrzyn and Ntambi, 2004). Liver and blood are two important metabolic transport systems that regulate lipid metabolism. In this study, we showed that serum TG, TC and insulin levels in fish fed the SFA diet were higher than those in fish fed the control diet. Increased levels of serum insulin would favor glucose uptake by the liver and consequently lead to better glycemic control (Qiang et al., 2016). We also found the 5% SFA diet did not cause the accumulation of fat droplets in hepatic structure, and no significant differences in serum ALT and AST levels were detected between fish fed the SFA diet and fish fed the control diet. These results suggested that SCD gene expression was regulated in GIFT fed the 5% SFA diet, and this in turn stimulated lipid transport in serum to maintain lipid metabolism homeostasis of liver. The SCD gene of GIFT is abundantly expressed in the liver. Therefore, from this study, we selected the tilapia liver as the subject of study to analyze the regulation of SCD by miR-29a and the changes of related indexes. However, organisms are interconnected systems, in which cells, tissues and organs work together. miR-29a regulation at different organizational levels may also induce synergies, so we will continue to investigate this issue in further studies.

In conclusion, miR-29a is induced by a SFA diet and regulates SCD by targeting the SCD 3′ UTR directly. The expression of miR-29a is downregulated in the liver of GIFT on day 60 of the SFA diet. miR-29a is involved in the regulatory circuit of fish liver through its role in the translational increase of SCD expression, which eventually regulates the signals related to fatty acid conversion and increases serum insulin to stimulate lipid and glucose transport. Overall, these results clearly suggest that miR-29a plays a crucial role in controlling lipid metabolism homeostasis of GIFT liver. Further investigations will focus on the functional mechanisms of miR-29a and SCD in lipid metabolism combined with analyses of their expression profiles in knock-out fish or in fish injected with antagomir antisense oligonucleotides *in vivo* and *in vitro*.
